# Inflammation as a Therapeutic Target in Cancer Cachexia

**DOI:** 10.3390/cancers14215262

**Published:** 2022-10-26

**Authors:** Gerald Clamon, Margaret M. Byrne, Erin E. Talbert

**Affiliations:** 1 Department of Internal Medicine, Division of Hematology/Oncology, Marrow and Stem Cell Transplantation and Cellular Therapy, University of Iowa Hospitals and Clinics, 200 Hawkins Drive, Iowa City, IA 52242, USA; 2 Department of Health and Human Physiology, University of Iowa, 285 Newton Road, Iowa City, IA 52242, USA

**Keywords:** cancer-associated weight loss, tissue wasting, cytokines

## Abstract

**Simple Summary:**

Weight loss, called cachexia, often occurs in cancer patients, and is associated with poor quality of life and shorter survival. Cancer cachexia is associated with increased circulating inflammatory factors, but efforts to reduce levels of inflammation using a single agent have not been successful. Cachexia likely results from many causes, including inflammatory cytokines, anorexia, catabolism, depression, and pain, and targeting the multiple causes will likely be necessary to achieve improvement in weight and appetite. In this review, we explore some potential therapies to address the need for anti-cachexia therapy with an emphasis on existing therapies that may be able to be re-purposed, therapies targeting inflammation, and the likely need for combination therapy directed at the complex causes of cancer cachexia.

**Abstract:**

Cachexia is a common complication of cancer and is associated with poor quality of life and a decrease in survival. Many patients with cancer cachexia suffer from inflammation associated with elevated cytokines, such as interleukin-1beta (IL-1β), interleukin-6 (IL-6), and tumor necrosis factor (TNF). Single-agent trials to treat cancer cachexia have not led to substantial benefit as the type of cytokine which is elevated has rarely been specified and targeted. Cachexia may also be multifactorial, involving inflammation, anorexia, catabolism, depression, and pain, and targeting the multiple causes will likely be necessary to achieve improvement in weight and appetite. A PUBMED search revealed over 3000 articles on cancer cachexia in the past ten years. We attempted to review any studies related to inflammation and cancer cachexia identified by Google Scholar and PUBMED and further search for articles listed in their references. The National Comprehensive Cancer Network (NCCN) guidelines do not provide any suggestion for managing cancer cachexia except a dietary consult. A more targeted approach to developing therapies for cancer cachexia might lead to more personalized and effective therapy.

## 1. Introduction

Cancer cachexia is defined as weight loss of greater than 5% in the 6 months prior to diagnosis or greater than 2% if the patient started with a body mass index (BMI) of 20 kg/m^2^ or less [1]. Cachexia may be seen in 30–50% of cancer patients and is associated with a poorer prognosis [2,3,4]. In addition to weight loss, cachexia often involves anorexia, catabolism with loss of muscle mass, and at times fever, which are all associated with decreased quality of life. Markers of inflammation such as C-reactive protein (CRP) and neutrophil/lymphocyte ratio (NLR) are generally elevated [5,6,7]. Even with aggressive supplemental nutrition, patients often experience continued signs and symptoms of cancer cachexia, as well as a continued negative nitrogen balance.

Loss of muscle mass is associated with poor quality of life and depression in patients with advanced cancer [8], and there is significant need for additional therapies to treat cachexia in cancer patients. In this review, we explore some potential therapies to address this need with an emphasis on existing therapies that may be able to be re-purposed as anti-cachexia therapies, therapy targeted at inflammation and cytokines, and the likely need for combination therapy directed at the complex metabolic causes of cancer cachexia.

## 2. Mechanisms of Cancer-Associated Cachexia

Cancer-mediated cachexia has long been thought to be mediated by increased circulating levels of inflammation [2]. Pro-inflammatory cytokines, such as tumor necrosis factor (TNF), interleukin-1beta (IL-1β), interleukin-6 (IL-6), and interferon gamma (IFN-γ), leading to activation of the Janus Kinase-signal transducer of transcription (JAK/STAT) pathway may be involved. A review on advances in cancer cachexia in 2020 emphasized the complexity of the various cytokines involved and the effects on various organs [9].

In addition to cytokine release, cachexia may also arise from potentially treatable causes of weight loss such as hyperthyroidism, depression, infection, diarrhea, bowel obstruction, impaired swallowing, esophageal obstruction, malabsorption, and secretory diarrhea. Further, as cancer frequently involves more elderly patients, loss of muscle mass may already be occurring due to the aging process [10].

Dysbiosis or abnormal gut microbiota are associated with an increased inflammatory state in patients with cancer cachexia [11]. In animal models, antibiotic therapy for the gut microbiota has led to some improvements in inflammation. Dysbiosis has also been associated with a poor response to immunotherapy with anti-programmed death (PD1) or anti-programmed death-ligand (PDL1) therapy. Herremans et al. have summarized the extensive literature on the gut microbiome and cancer cachexia [11].

Although the weight loss associated with cancer cachexia is most often discussed in terms of loss of muscle mass, there is also a loss of fat storage and depletion of adipose tissue [12]. This is associated with changes in adipokines, such as leptin, adiponectin, and resistin, which modulate inflammation. The adipokine alterations may not be the initiating factor for cancer cachexia but may exacerbate the inflammatory state produced with cancer cachexia.

## 3. Current Clinical Management

The optimal management of cancer cachexia is not well-defined. The American Society of Clinical Oncology (ASCO) guidelines for management of cancer cachexia published in 2020 recommend consultation with a dietician [13]. Nutritional support, such as hyperalimentation, has not improved overall survival in patients with metastatic solid tumors despite providing adequate calories. In the absence of curative therapy for a specific cancer, it is not likely that nutritional support will have a huge impact on either disease-free survival or overall survival. In the recent past, the Food and Drug Administration (FDA) included improvement in strength as a requirement for the approval of an agent in the management of cancer cachexia. An increase in lean body mass and improvement in strength likely require a concurrent exercise program and longer anticipated survival than a few months [14]. Lambert has questioned whether the clinical efficacy of cachexia drugs should depend on improvement in strength given limitations in this measure [14].

The 2020 ASCO guidelines do not recommend using any pharmacologic agents for the treatment of cancer cachexia, citing a lack of evidence, consistent with ASCO education books. Neither the guidelines nor the ASCO education book discuss the role of inflammation and cytokines, such as IL-1β, IL-6, JAK/STAT pathway, or IFN-γ, all of which have been associated with cancer cachexia. In the ASCO guidelines, TNF inhibition is discussed, although its use is not recommended [13]. Trials of anamorelin for cachexia in non-small cell lung cancer (NSCLC) and enobosarm, an androgen receptor modulator, have shown promise but have not yet achieved FDA approval in the US. Anamorelin is approved in Japan based on randomized trials showing an improvement in lean body mass compared with control patients, but it did not show improvement in handgrip strength [15,16]. Additional clinical trials are underway to investigate quality-of-life (QOL) in patients treated with anamorelin (NCT03743064 and NCT03743051). Management of cancer cachexia has often been attempted with megestrol acetate, although recent evidence suggests it is ineffective with significant side effects [17]. Similarly, agents such as glucocorticoids, anti-depressants, or anabolic steroids have been used off-label [17,18,19].

Because of the complex etiology of cancer cachexia and the failure to address the need for targeted therapy, single-agent pharmacologic therapy has not been effective. While personalized care directed at abnormal mutations is now standard for the treatment of cancer, there has not been a personalized approach to the management of cancer cachexia.

What should be the goal of an effective anti-cachexia therapy? In our approach to supportive care, we do not demand that effective pain medications prolong survival or improve strength. Relief of pain and improved QOL are acceptable goals. For patients with incurable, advanced malignancies, improvement in QOL with therapy for cancer cachexia should be an adequate goal for approval by the FDA. If a cancer cachexia trial were to be done in NSCLC, QOL could be measured by the Functional Assessment of Cancer Therapy- Lung (FACT-L) questionnaire, which has been validated in lung cancer and for which the endpoints to definite success have been defined [20]. Future trials in lung cancer patients with earlier stage disease and cancer cachexia may be performed using additional endpoints beyond QOL, such as lean body mass, overall survival, response to therapy, and improvement in strength in patients capable of exercise training.

**Figure 1:**
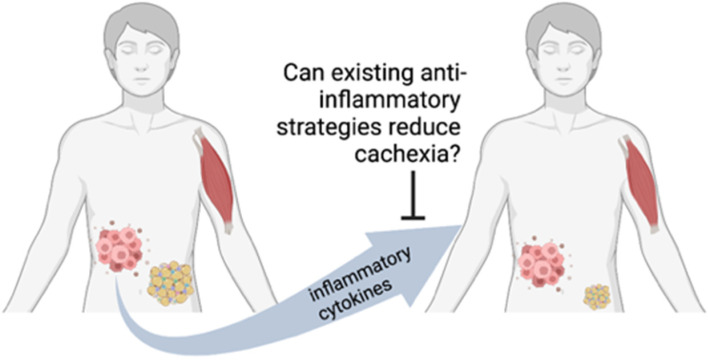
Review question. Created with Biorender.com on 10 October 2022.

We sought to review the existing clinical trial literature, including some pre-clinical data that led to clinical trials. We had a particular interest in compounds currently in use for other indications that could be potentially repurposed for use as an anti-cachexia therapy, which led us to question if existing anti-inflammatory strategies might be effective to prevent cancer cachexia (Figure 1). 

## 4. Search Strategy

Searches were made on PUBMED and GOOGLE Scholar covering the years from 2000 to 2021 and then during revision studies including 2022 were added. Searches were made including: (1) Cancer cachexia, (2) cancer and weight loss, (3) cancer cachexia and interleukin-1β, (4) cancer cachexia and interleukin-6, (5) cancer cachexia and TNF, (6) cancer cachexia and JAK2, (7) cancer and sarcopenia. There are thousands of articles over the past 20 years that came up with these searches and those whose title and abstract did not appear relevant were excluded as were multiple studies by the same authors where the most recent studies updated prior information. Separate searches were made for omega-3 fatty acids and anamorelin which have been proposed for treatment of cancer cachexia. Articles were selected based on the title, abstract and then review of the entire manuscript. The searches for IL-1β, IL-6, TNF, and JAK were based on reports that these were involved in cancer cachexia syndromes. The authors examined and added references and text based on their area of expertise.

## 5. Current Status of Anti-Inflammatory Strategies for Cancer Cachexia

Because of the highly inflammatory nature of cancer, there has long been an interest in the use of anti-inflammatory medications to prevent cancer cachexia. In 2013, a systematic review of randomized clinical trials of nonsteroidal anti-inflammatory drugs suggested these agents show promise in improving QOL [21]. However, this review concluded that insufficient evidence exists to recommend these agents broadly, primarily because only four clinical trials met the review inclusion criteria. In this section, we review relevant pre-clinical studies, the four trials reviewed in Reid et al. [21], and new randomized clinical trials that have been conducted since that time.

### 5.1. Fish Oils and Omega-3 Fatty Acid Supplements

Because of their broad anti-inflammatory properties including reductions in prostaglandin and leukotriene synthesis [22], many studies of omega-3 fatty acid supplements or fish oils for cancer cachexia have been conducted. A recent review summarized the clinical trials of omega-3 fatty acids that have been conducted and concluded that the results have inconsistently shown benefit for cancer patients [23]. Two studies of particular interest are highlighted here. First, a Cancer and Leukemia Group B (CALGB) trial initiated at the University of Iowa [24] demonstrated improvement in weight loss in some patients if therapy was continued for more than one month but a trial from MD Anderson found that a 2-week course of omega-3 fatty acids was of no benefit [25].

### 5.2. Non-Steroid Anti-Inflammatory Drugs

Non-steroidal anti-inflammatory drugs (NSAIDs) have long been considered as a therapy for cancer cachexia because of their strong safety profile, good tolerability, and broad-spectrum effects. Below, relevant pre-clinical studies and clinical trials involving NSAIDs in cancer cachexia are reviewed. Key clinical trials for NSAIDs in cancer cachexia that were identified are summarized in Table 1.

#### 5.2.1. Celecoxib

Celecoxib is believed to reduce prostaglandin synthesis via specific inhibition of cyclooxygenase-2 (COX-2) [33]. Mantovani et al. reported a phase II nonrandomized trial of celecoxib in 24 patients with cancer cachexia treated with celecoxib 300 mg/day for 4 months [26]. From baseline to end of treatment, there was a non-significant change in levels of IL-6, but there was a significant drop in TNF. There was also a significant improvement in QOL and Eastern Cooperative Oncology Group (ECOG) performance score. Lai et al. reported a pilot study with only 11 patients with cancer cachexia due to cancer of the head and neck or GI tract treated with celecoxib [27]. Patients were randomized to placebo vs. celecoxib 200 mg bid. Although the conclusion of this manuscript was that QOL was improved, this was not a significant change as there were too few patients. Levels of IL-6 and IL-8 improved with celecoxib vs. control but there were so few patients that none of the changes were statistically significant.

Five trials have been reported with the combinations of celecoxib and other agents for cancer cachexia [28,29,30,34,35]. Madeddu et al. [34] randomized 60 patients with a variety of cancers to receive either carnitine plus celecoxib or carnitine plus celecoxib plus megestrol acetate. All patients were also supplemented with polyphenols, lipoic acid, carbocysteine, vitamin E, Vitamin A, and vitamin C. The dose of celecoxib was 300 mg/day. The two-drug regimen was not inferior to the 3-drug regimen with respect to lean body mass and 6-min walk test. There were no significant changes in QOL in either treatment arm. 

Kouchaki et al. [28] compared megestrol with placebo vs. megestrol and celecoxib in a randomized trial with 90 patients. The dose of celecoxib was 200 mg/day, which is less than the dose in other trials. After one month of therapy, 60 patients were evaluable, and after two months 33 were evaluable. The two-month data showed no difference between the groups with respect to weight gain, though both were improved over baseline. Secondary measures including grip strength, albumin, and IL-6 were not different between groups. The number of evaluable patients was as low as 10 in each treatment arm, limiting the study’s power to find statistically significant differences between the two groups. However, the appetite score improved more for the placebo group than the combination arm. Quality of life was improved in both treatment groups.

Cerchietti et al. [35] evaluated 15 lung cancer patients with “systemic immune metabolic syndrome” defined in the study as cachexia, anorexia, nausea, early satiety, fatigue, tumor fever, cognitive changes, and/or infection. Therapy was with celecoxib 200 mg bid, medroxyprogesterone 500 mg bid. While this was a small pilot study, 13 patients had stable or improved weight. Quality of life was not measured.

In another study by the same group [29], in an initial cohort of patients, 6 gm of fish oil (eicosapentaenoic and docosahexaenoic n-3 fatty acids) per day was determined to be the maximally tolerated dose. Then, 22 patients were randomized to fish oil (2 gm tid) and placebo vs. fish oil (2 gm tid) plus celecoxib 200 mg bid. After 6 weeks, subjects in the fish oil plus celecoxib group had significantly lower levels of CRP, higher muscle strength, and improved body weight. Although patients reportedly had symptom improvement, there were no quality-of-life measures for this trial.

Solheim et al. [30] studied 46 patients with lung cancer or pancreatic cancer with cancer cachexia. Notably, to enroll the 46 patients, they had to screen 399 patients. All patients had two cycles of chemotherapy and then were randomized to either standard care vs. celecoxib 300 mg per day, 2 cartons of Prosure to provide 2 gm/day of omega-3 fatty acid, and an exercise program. Forty-one of the 46 patients completed the study. Compliance with celecoxib was 76%, exercise was 60%, and nutritional supplement was 48%. Those in the treatment arm gained weight of 1.29% while those in the control arm lost 3.19%. Both the control arm and the treatment arm showed loss of muscle mass, but the loss was less in the treatment group. They used an assessment called PG-SGA which was an abridged patient generated subjective global assessment. There was no difference in the quality-of-life measure between the control and the treatment groups.

#### 5.2.2. Ibuprofen

Ibuprofen is a non-specific inhibitor of COX enzymes, and therefore is also believed to reduce prostaglandin synthesis [36]. Animal models of cancer cachexia treated with ibuprofen have yielded inconsistent results. In mice with a colon-26 implant, ibuprofen improved body weight and weight of the gastrocnemius muscle [37]. On the other hand, in Walker 256 tumors in rats, celecoxib prevented loss of muscle mass and the rise in blood levels of urea and lactate and attenuated the decrease in food intake, but ibuprofen did not [38]. A randomized clinical trial of 73 patients with metastatic gastrointestinal cancers began with 38 patients randomized to megestrol/placebo and 35 to megestrol/ibuprofen [31]. The median on-study weight loss was 18%, and 46 of the 73 patients were unable to complete 12 weeks of study therapy, mainly due to progression of cancer. The megestrol dose was 160 mg tid and the ibuprofen dose was 400 mg tid. Of the patients who completed 12 weeks of therapy, those on megestrol alone showed a median weight loss of 2.8 kg while those on megestrol and ibuprofen had a median weight gain of 2.3 kg.

#### 5.2.3. Naproxen

Similar to ibuprofen, naproxen is an inhibitor of COX enzymes believed to reduce prostaglandin synthesis [36]. Two trials in cancer-bearing animals treated with naproxen along with other agents showed reduced tumor growth [39,40]. Naproxen alone increased weight by 14% in rats with Walker 256 tumors, and the combination of naproxen, clenbuterol, and insulin increased weight by 41% compared with controls. Rats with Walker 256 tumors treated with fish oil and then supplemented with fish oil along with the combination of naproxen, clenbuterol and insulin decreased tumor growth and cachexia. No clinical trials were identified with naproxen for cancer cachexia. 

#### 5.2.4. Indomethacin

Similar to the other NSAIDs discussed, indomethacin is believed to reduce prostaglandin synthesis through COX enzyme inhibition [41]. Indomethacin has been studied in animal models of cancer cachexia [42,43,44,45]. Diament et al. studied mice with LPO7 lung adenocarcinoma with medroxyprogesterone acetate (MPA) and indomethacin [42]. The combination inhibited tumor growth, although single-agent MPA did not. There was a decrease in IL-6 and metalloproteinases with the combination. Leukocytosis and cachexia were reduced with both agents. Lonnroth et al. [43] found that mice with a malignant melanoma cell line treated with indomethacin had no improvement in survival, nutritional status, or tumor growth. In an epithelial tumor-bearing mouse (MCG101), there was a hundred-fold increase in prostaglandins. This was inhibited by indomethacin and led to a prolongation of tumor doubling time [43]. Chen and Qiu [44] found that a combination of growth hormone, insulin, and indomethacin relieved cancer cachexia in a mouse model. Xu et al. [45] reported in an abstract that C57 mice with Lewis lung carcinoma treated with indomethacin had a downregulation of IL-1β, IL-6, and TNF. This therapy also prolonged survival in this mouse model.

A clinical trial of indomethacin found that Karnofsky performance score was preserved in treated patients, while Karnofsky performance score declined during the treatment period in placebo-treated patients [32]. Indomethacin-treated patients also had improved survival compared to placebo-treated patients.

### 5.3. Targeted Anti-Inflammatory Strategies

Circulating inflammatory cytokines have long been thought to be the primary cause of muscle wasting in cancer patients, and a number of studies have been conducted to specifically target individual cytokines. For most of these studies, individual patients were not screened for elevated levels of the target cytokine prior to therapy initaition, but newer trials have begun to include elevated levels of the cytokine in the inclusion criteria.

#### 5.3.1. TNF

Argiles et al. presented a very thorough review of the newer agents proposed for treating cancer cachexia, including TNF inhibitors [46]. TNF is elevated in some patients with cancer cachexia, and it was hoped that various agents inhibiting TNF would be effective treatment. However, trials with thalidomide, etanercepts, or infliximab did not consistently show benefit [46].

#### 5.3.2. IL-1β

A recent review of the role of IL-1β in cancer cachexia by Laird et al. [47] reports that elevated IL-1β leads to increased muscle and adipose loss, leading to anorexia and increased energy expenditure. Neuro-inflammation associated with IL-1β via the pituitary axis leads to increased muscle proteolysis and lipolysis. Laird et al. argue that the pathophysiology of cancer cachexia suggests that canakinumab, an IL-1β inhibitor might be a viable therapy.

#### 5.3.3. IL-6

IL-6 may be associated with muscle wasting in animal models of cancer cachexia [48]. Inhibition of STAT3 via the JAK/STAT pathway or STAT3 inhibitors may reduce cachexia in patients suffering from elevated IL-6. Tocilizumab, an available inhibitor of IL-6 might be an effective agent for patients with elevated levels of IL-6. Using a mouse model of cancer cachexia, Guo et al. [49] were able to reduce weight loss and muscle wasting in tumor bearing animals with pantoprazole. Pantoprazole therapy can lower both IL-6 (56%) and TNF (67%) to some extent in this animal model. However, animal models may not be predictive of results in humans [50,51].

Elevated levels of IL-6 have been associated with a poor prognosis [52]. In a mouse model, tocilizumab, an IL-6 inhibitor, improved cachexia [53]. Hirata et al. [54] reported 2 cases of successful treatment with tocilizumab in patients with cancer cachexia. In one patient, tocilizumab 8 mg/kg given IV resolved tumor fever within one day of therapy initiation and led to improved appetite and dietary intake. The serum albumin improved as did the Hgb, and the CRP decreased. However, these improvements were short-lived lasting, only 2–3 weeks. A second patient with 11.7% weight loss, anemia, and tumor fever responded to tocilizumab after failing to improve on naproxen and prednisone. He had a 12.7% weight gain while on tocilizumab with improvement in his Hgb. Some of the same physicians also reported a third case of cancer cachexia, which dramatically improved with tocilizumab [55].

Activation of the JAK/STAT pathway, which is downstream from IL-6, has been associated with cancer cachexia. Two JAK1/JAK2 inhibitors have been considered for cancer therapy. Ruxolitinib shows anti-inflammatory efficacy in myelofibrobrosis with improved quality of life [56] and has progressed to a clinical trial (NCT04906746). Pacritinib has been studied in myeloproliferative disease [57] and may also be an option for cancer cachexia.

## 6. Other Anti-Cachexia Strategies of Note

A recent review from Talbert and Guttridge highlighted the current status of targeting four additional probable mediators of cancer cachexia - activin A, myostatin, GDF15, and lipocalin-2 [58]. We present two additional potential avenues for anti-cachexia treatment.

### 6.1. Immune Checkpoint Inhibitors

Retrospective studies demonstrate that cachexia prior to any treatment in patients with lung cancer or other cancers is associated with poor response to immune checkpoint inhibitors [59]. This may be due to elevated immune checkpoint inhibitor clearance [60].

Degens et al. [59] reported on weight loss in 106 patients with NSCLC treated with nivolumab and an additional 62 patients in a validation cohort. Patients who had weight loss of >2% after 6 weeks of therapy had worse survival. In the initial cohort, 34% of patients had weight loss in the first 6 weeks, and in the validation cohort 45% of patients had weight loss at 6 weeks. In both cohorts, the median survival was about 6 months for patients with weight loss and over 18 months in patients without weight loss.

Jo et al. [2] reported on 133 patients with NSCLC treated with pembrolizumab. At baseline, 35% of patients had cachexia using the standard definition and median survival was 10 months vs. 26 months in those patients presenting without cachexia. IL-1α, TNF, IL-8, and IL-10 were significantly elevated in cachexia patients but IL-1β and IL-6 were not significantly elevated in the cachexia patients. Leptin was significantly higher in the non-cachexia patients. Similarly, Roch et al. [61] reported that among 142 patients with NSCLC treated with immune checkpoint inhibitors, those with > 5% weight loss at baseline or those with sarcopenia has significantly shorter median survival than patients not suffering from weight loss or sarcopenia. Guzman-Prado et al. [62] identified 7 studies in a review of sarcopenia and the risk of side effects in cancer patients treated with immune checkpoint inhibitors. In their review, they concluded that the presence of sarcopenia at baseline was associated with increased risk of adverse effects from immune checkpoint inhibitors.

### 6.2. MEK Inhibition

The potential anti-cachectic activity of MEK inhibitors was initially suggested by the finding of weight gain in patients with cholangiocarcinoma treated with the MEK inhibitor, selumetinib [63,64]. Despite a lack of anti-tumor activity, patients gained weight, and a further analysis suggested that the majority of the weight gain was due to a gain of muscle mass. Talbert et al. [65] used animal models of cancer cachexia with colon-26 to study MEK inhibition with MEK162. Talbert found that inhibition of MEK and also PI3K/AKT with buparlisib demonstrated both anti-tumor activity and improved cachexia. Au et al. [66] showed that the MEK inhibitor selumetinib decreased tumor growth and reduced IL-6 but did not inhibit muscle wasting in the Lewis Lung cancer model of cancer cachexia. This suggests there is more to be learned about the potential role of MEK inhibition in the treatment of cancer cachexia.

## 7. Conclusions

At this time, there are no recommendations for a specific agent or combination of agents for the management of cancer cachexia. A review on advances in cancer cachexia in 2020 emphasized the complexity of the various cytokines involved and the effect on various organs including data in both animal trials, as well as human trials [9]. Although a systematic review in 2012 suggested that NSAIDS show promise in improving quality of life, the conclusion was that these agents have been insufficiently studied [21]. It is possible that clinical trials based on elevated biomarker levels (such as IL-1β, IL-6, or TNF) in each individual patient will allow a more targeted approach to ameliorating cancer cachexia. Further, combinations of anti-inflammatory agents with appetite stimulating agents such as anamorelin and anabolic steroids may be more effective than single-agent therapy. Presently, an improvement in quality of life should be an appropriate endpoint for drug approval in the majority of these patients with a terminal cancer. In addition, demonstrating a positive nitrogen balance and/or improvement in retinol binding protein may prove to be useful intermediate endpoints as improving muscle mass may depend on a longer survival than many patients will experience.

## Figures and Tables

**Table 1:** NSAIDs in anti-cachexia clinical trials.

Compound	Trial Details	Key Findings in NSAID Group	Reference
Celecoxib	Single arm Phase II; 24 patients; 300 mg/day; 4 months.	~1% ↑ LBM (*p* < 0.0001) 19% ↓ TNF (*p* = 0.007) 15% ↑ HGS (*p* = 0.004) 14% ↑ QOL (*p* = 0.024) 20% ↓ ECOG (*p* = 0.0004)	[26]
	Randomized pilot; 11 patients; 200 mg bid; 21 days	Likely benefit, but low power 1.4% ↑ weight (*p* = 0.05) ↑ QOL (*p* = 0.05) 19% ↓ IL-6 (*p* = 0.18) 33% ↓ IL-8 (*p* = 0.19)	[27]
	Phase III double-blind randomized; 90 patients; megestrol acetate + placebo v. megestrol + 200 mg/day celecoxib; 30 days	No additional benefits of celecoxib	[28]
	Randomized; 22 patients; fish oil (2 gm tid) + placebo v. fish oil (2 gm tid) + celecoxib 200 mg bid; 6 weeks	55% ↓ CRP (*p* = 0.05) 15% ↑ HGS (*p* = 0.02) 2.5% ↑ body weight (*p* = 0.05)	[29]
	Phase II randomized; 46 patients; standard care v. celecoxib 300 mg/ day + 2 gm/day omega-3 fatty acid + exercise; 6 weeks	4.48% ↑ body weight (*p* < 0.001) ↑ muscle mass (*p* = 0.03)	[30]
Ibuprofen	Randomized; 73 patients; megestrol + placebo v. megestrol + ibuprofen 400 mg tid; 12 weeks	2.3 kg ↑ body weight (*p* < 0.001)	[31]
Indomethacin	Randomized; 135 patients; 50 mg bid; variable length	14% ↑ KPS (*p* = 0.03) ↑ survival (*p* < 0.05)	[32]

↑ = increased, ↓ = decreased, LBM = lean body mass, HGS = hand grip strength, KPS = Karnofsky performance score.
